# Prognosis and postoperative surveillance of benign ovarian tumors in children: a single-center retrospective study

**DOI:** 10.1016/j.jped.2025.03.009

**Published:** 2025-05-10

**Authors:** XiaoLi Chen, DuoTe Cai, Yi Chen, Weiwei Chen, YueBin Zhang, ZhiGang Gao, QingJiang Chen

**Affiliations:** Zhejiang University School of Medicine, The Children’s Hospital, National Clinical Research Center For Child Health, Department of General Surgery, Zhejiang, China

**Keywords:** Pediatric, Benign ovarian tumor, Prognosis, Follow-up

## Abstract

**Objective:**

To evaluate the prognosis of benign ovarian tumors and develop a postoperative surveillance strategy for children based on the findings.

**Methods:**

The clinical data of children with benign ovarian tumors treated in the hospital from January 2014 to December 2021 were retrospectively analyzed.

**Results:**

A total of 404 patients were included in this study, with an average age of 9.1 ± 3.1 years. All patients underwent a total of 423 procedures, including 61 oophorectomy and 362 ovary-sparing surgeries. 67 patients were lost to follow-up after surgery. The remaining 337 patients were followed up for a period ranging from 3 months to 9 years (mean 1.6 ± 1.8 years). The ovarian preservation rate for patients undergoing ovary-sparing surgery for the first time was 94.4% (271/287). The overall recurrence rate of benign ovarian tumors was 3.9% (13/337). Of the 13 patients with recurrence, 10 had regular imaging examinations and did not develop symptoms. Three patients had irregular follow-up after surgery and returned to the hospital due to symptoms. The first recurrence interval of these 13 patients after surgery ranged from 0.6 to 5.3 years (mean 2.0 ± 1.4 years). 84.6% (11/13) of the recurrence cases developed within 3 years after surgery.

**Conclusion:**

Ovary-sparing surgery for benign ovarian tumors has a favorable prognosis and a high rate of ovarian preservation. Regular follow-up after surgery for benign ovarian tumors is necessary. Annual imaging follow-up for at least 3 years postoperative can detect most recurrence cases.

## Introduction

Ovarian tumors in children are rare, with an estimated incidence of 2.2/100,000 [1]. Most ovarian tumors are benign, and malignant tumors account for approximately 3.7% −25% [2]. Ovary-sparing surgery is safe and effective, which is the first choice for benign tumors [3,4]. Despite the benign nature of the tumor, many articles have reported the postoperative occurrence of ipsilateral recurrence and contralateral metachronous ovarian tumors [5,6]. The overall incidence rate of contralateral metachronous ovarian tumors is 2.1% [7]. However, there is currently no consensus on postoperative surveillance strategies.

A survey of surgeons demonstrates that follow-up of patients is primarily guided by individual protocols, with wide variation among surgeons in frequency, duration, and further investigations during the follow-up period [8]. Some authors believe that follow-up for benign ovarian tumors should last at least six months [6], while others recommend extending the follow-up period until the age of 16 [9] or until the patient's first pregnancy [10]. In a multicenter retrospective study, the authors argue that routine imaging for asymptomatic patients not only fails to assist in identifying malignant lesions but also increases healthcare costs and patient/family burden [11]. Therefore, they support performing symptomatic imaging postoperatively rather than routine imaging for patients with benign ovarian tumors. To date, large-scale studies on this topic remain scarce, and there is still a lack of strong, evidence-based guidance.

The purpose of this study is to evaluate the prognosis of benign ovarian tumors, including ovarian preservation rate and recurrence rates (both ipsilateral and contralateral), by retrospectively analyzing clinical data of a large sample of pediatric patients in a single center. To explore the postoperative surveillance strategy for benign ovarian tumors in children based on the findings.

## Materials and methods

This study was approved by the Ethics Committee of the present study’s institution (2024-IRB-0322-P-01).

The clinical data of children with benign ovarian tumors treated in the hospital from January 2014 to December 2021 were retrospectively analyzed. All patients underwent surgery for ovarian tumors for the first time, and postoperative pathology indicated benign ovarian tumors. Patients with pathologically confirmed malignant tumors or non-neoplastic ovarian lesions were excluded. Collect patients' medical records, including age, surgical method, intraoperative findings, postoperative pathology, and follow-up. All data were analyzed by Excel software.

Laparoscopy is the primary surgical approach. During ovary-sparing surgery, for small tumors, the ovarian parenchyma is incised to remove the tumor, followed by suturing and reconstructing the ovary. For large tumors, a small incision is first made in the umbilicus, the cyst is punctured and decompressed, and then the ovary is exteriorized through the umbilical incision. After the tumor is removed, the ovary is repositioned back into the pelvic cavity. Suture the umbilical incision, establish pneumoperitoneum, and explore the size and morphology of the contralateral ovary and uterus under laparoscopy.

The surveillance strategy is a routine ovarian ultrasound before discharge and another examination 3 months after discharge. There is no standardized protocol for surveillance after 3 months, and the duration and frequency of follow-up depend on each surgeon's personal preferences. Definition of surgical failure: the identification of ipsilateral lesions on imaging within 12 weeks after the initial surgery, which has the same pathology as the initial surgery; the detection of contralateral lesions on imaging within 12 weeks after the initial surgery, which are pathologically confirmed benign ovarian tumors. Radiologically suspected failure of surgery refers to surgical failure that only has imaging evidence without pathological verification. Definition of recurrence: the identification of lesions on the ipsilateral or contralateral ovarian by imaging more than 12 weeks after the initial surgery, which has the same pathology as the initial surgery. Radiologically suspected recurrence refers to a recurrence that only has imaging evidence without pathological verification. In this study, the term 'prognosis' specifically refers to oncologic outcomes, particularly recurrence rates in patients with benign ovarian tumors.

## Results

A total of 404 patients were included in this study, with an average age of 9.1 ± 3.1 years. Twenty patients had bilateral lesions, including 14 cases with synchronous bilateral lesions and 6 cases with metachronous bilateral lesions. Laparoscopy was performed in 401 cases, of which 2 cases were converted to open surgery. The remaining 3 cases underwent open surgery. All patients underwent a total of 423 procedures, including 61 oophorectomy and 362 ovary-sparing surgeries. There were 99 cases of ovarian torsion identified during surgery and 2 cases with more than 2 lesions in a single ovary. 67 patients were lost to follow-up after surgery. The remaining 337 patients were followed up for a period ranging from 3 months to 9 years (mean 1.6 ± 1.8 years) (Table 1).

**Table 1:** Full cohort characteristics (*n* = 404).

Patient characteristics	n	%
Surgical age(y)	9.1 ± 3.1	
Side		
Unilateral	384	95.0%
Synchronous bilateral	14	3.5%
Metachronous bilateral	6	1.5%
Index procedure		
Ovary-sparing surgery	343	84.9%
Oophorectomy	61	15.1%
Index pathology		
Mature teratoma	342	84.7%
Serous cystadenoma	41	10.1%
Mucinous cystadenoma	21	5.2%
Follow-up period		
3M-1y	178	44.0%
1–2y	56	13.9%
2–3y	41	10.1%
3–9y	62	15.4%
Loss to follow-up	67	16.6%
Reoperation		
Failure of initial surgery	2	0.6%
Recurrence	13	3.9%
New lesion	1	0.3%

**Figure 1:**
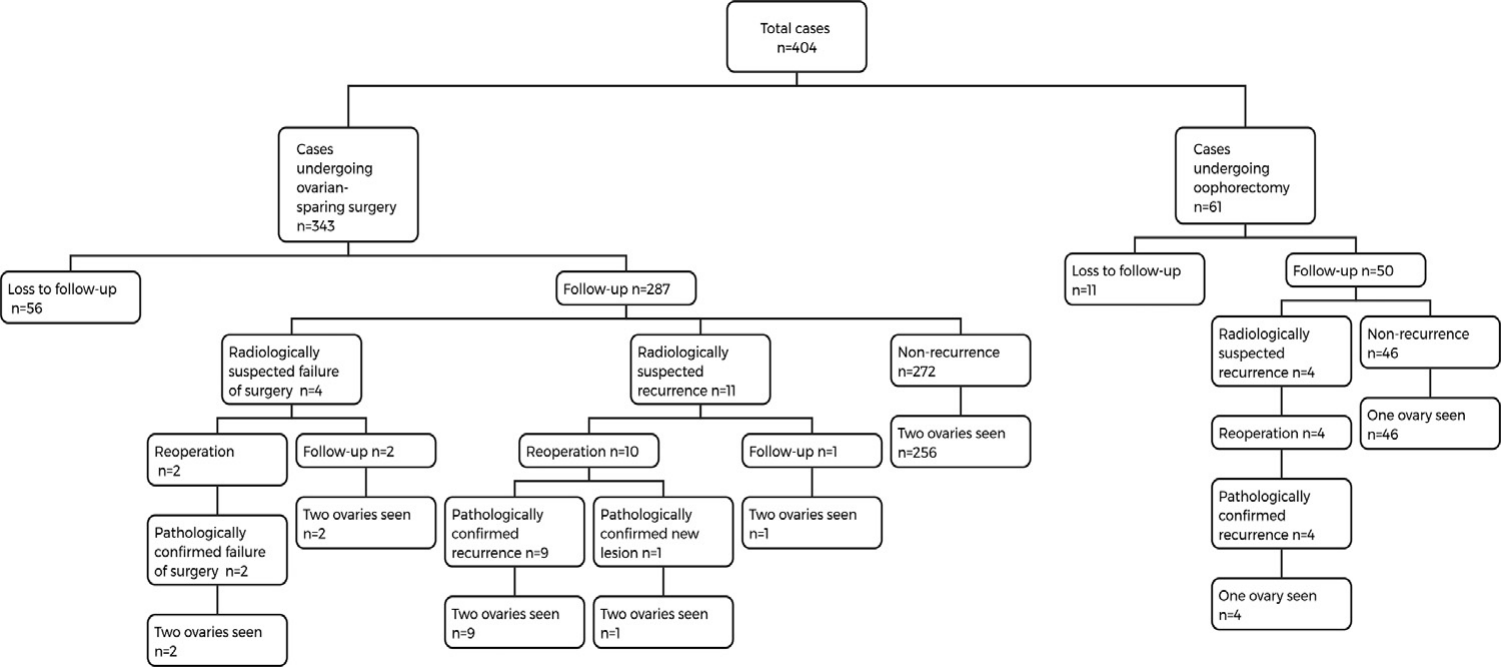
Prognosis of 404 patients with benign ovarian neoplasms.

Postoperative ultrasound revealed contralateral lesions in 4 cases before discharge, and the radiologist diagnosed the lesions as mature teratoma. Two patients underwent reoperation, and pathology confirmed mature teratomas in both cases. The other two cases chose observation and are still under follow-up. During follow-up, there were 15 cases suspected of recurrence by imaging, of which 14 underwent reoperation. Postoperative pathology confirmed recurrence in 13 cases and new lesions in 1 case (Figure 1). The overall recurrence rate of benign ovarian tumors was 3.9% (13/337).

Of the 13 patients with recurrence, 10 had regular imaging examinations and did not develop symptoms. Three patients had irregular follow-up after surgery and returned to the hospital due to symptoms. The first recurrence interval of these 13 patients after surgery ranged from 0.6 to 5.3 years (mean 2.0 ± 1.4 years). Most patients experienced a single tumor recurrence after surgery, while one patient had up to three recurrences (Table 2). Four patients with recurrence were treated with oophorectomy for the first time and ovary-sparing surgery for the second time.

**Table 2:** The clinical data of 16 patients undergoing reoperation.

Case		Surgical age (1st)	Surgical method (1st)	Pathology (1st)	First recurrence interval	Surgical age(2nd)	Surgical method (2nd)	Pathology (2nd)	Surgical age (3rd)	Surgical method (3rd)	Pathology (3rd)	Surgical age (4th)	Surgical method (4th)	Pathology (4th)
Recurrence case														
	1	3.4y	Left oophorectomy	Left mature teratoma	4.2y	7.6y	Ovary-sparing surgery	Right mature teratoma	10.5y	Ovary-sparing surgery	Right mature teratoma			
	2	10.9y	Right Oophorectomy	Right mature teratoma	1.1y	12.8y	Ovary-sparing surgery	Left mature teratoma						
	3	8.6y	Ovary-sparing surgery	bilateral mature teratoma	2y	10.7y	Ovary-sparing surgery	bilateral mature teratoma	12.1y	Ovary-sparing surgery	Left mature teratoma	15.1y	Ovary-sparing surgery	bilateral mature teratoma
	4	15.2y	Left Oophorectomy	Left mature teratoma	1.2y	16.4y	Ovary-sparing surgery	Right mature teratoma						
	5	8.8y	Ovary-sparing surgery	bilateral mature teratoma	2.6y	15.9y	Ovary-sparing surgery	Left mature teratoma						
	6	9.1y	Ovary-sparing surgery	Right mature teratoma	1.9y	11.1y	Ovary-sparing surgery	Right mature teratoma						
	7	7.2y	left oophorectomy	Left mature teratoma	5.3y	12.5y	Ovary-sparing surgery	Right mature teratoma						
	8	11.6y	Ovary-sparing surgery	Left serous cystadenoma	0.6y	13.0y	Ovary-sparing surgery	Left serous cystadenoma						
	9	9y	Ovary-sparing surgery	Left mature teratoma	1.7y	11.4y	Ovary-sparing surgery	Left mature teratoma						
	10	6.8y	Ovary-sparing surgery	Left mature teratoma	1.1y	8.6y	Ovary-sparing surgery	Left mature teratoma						
	11	9.3y	Ovary-sparing surgery	Left mature teratoma	1.5y	10.9y	Ovary-sparing surgery	Right mature teratoma						
	12	9.9y	Ovary-sparing surgery	Left mature teratoma	1.6y	12.2y	Ovary-sparing surgery	Right mature teratoma						
	13	9.6y	Ovary-sparing surgery	Left mucinous cystadenoma	0.6y	10.4y	Ovary-sparing surgery	Left mucinous cystadenoma						
Non-recurrence case														
	14	8.7y	Ovary-sparing surgery	Left mature teratoma	–	11.2y	Ovary-sparing surgery	Left follicular cyst						
Surgical failure case														
	15	8.9y	Ovary-sparing surgery	Left mature teratoma	–	11.6y	Ovary-sparing surgery	Right mature teratoma						
	16	13.6y	Ovary-sparing surgery	Right mature teratoma	–	13.8y	Ovary-sparing surgery	Left mature teratoma						

Pathological examination after the first surgery revealed 342 cases of mature teratoma, 41 cases of serous cystadenoma, and 21 cases of mucinous cystadenoma. After the second surgery, pathological examination revealed 11 cases of mature ovarian teratoma, 1 case of serous cystadenoma, and 1 case of mucinous cystadenoma. One new lesion was confirmed as a follicular cyst by pathology (Table 2).

During the follow-up period, non-neoplastic ovarian cysts were detected in 30 patients by ultrasound. Among these, 15 cysts resolved spontaneously, while the other 15 are still under follow-up. The ovarian preservation rate for patients undergoing ovary-sparing surgery for the first time was 94.4% (271/287).

## Discussion

This study retrospectively analyzed the data of pediatric patients with benign ovarian tumors treated in the hospital over the past 8 years, revealing that the ovarian preservation rate of ovary-sparing surgery was 94.4%, and the recurrence rate was 3.9%. Among the 13 patients with recurrence, 10 cases (77%) were identified through regular follow-up, which underscores the importance of consistent postoperative follow-up. 84.6% (11/13) of the recurrence cases developed within 3 years after surgery. Therefore, annual imaging follow-up for at least 3 years postoperative can detect most recurrence cases.

Oophorectomy can lead to premature ovarian insufficiency, early menopause, decreased bone density, and increased risks of cardiovascular disease [12,13]. Additionally, oophorectomy does not prevent the risk of metachronous contralateral ovarian tumor recurrence or the development of new lesions [14,15]. Therefore, oophorectomy is no longer recommended for benign ovarian tumors in children. Knaus et al. reported a case who underwent oophorectomy for mucinous cystadenoma and eventually developed a mucinous tumor of low malignant potential in the contralateral ovary, requiring further oophorectomy [16]. In this study, four patients experienced contralateral recurrence after the first oophorectomy. Pathology from the second, ovary-sparing surgery confirmed all cases to be benign tumors. Consistent with other studies [17], the authors found favorable outcomes following ovary-sparing surgery, with an ovarian preservation rate of 94.4%. Nevertheless, there are still reports indicating that oophorectomy remains common for benign ovarian tumors, particularly in rural hospitals [18]. Encouragingly, Minneci et al. [13] reduced unnecessary oophorectomies from 16.1% to 8.4% in a multicenter study by using a preoperative risk stratification algorithm to identify lesions with a high likelihood of being benign and suitable for ovary-sparing surgery.

In this study, multiple ipsilateral ovarian teratomas during surgery and repeated recurrences of ovarian teratomas postoperatively were detected, which has been reported in other literature [14,19,20]. Sinha et al. [19]. reported a rare case where a patient was found to have seven dermoid cysts in the left ovary and two dermoid cysts in the right ovary during surgery. Pepe et al. [20]. reported a woman with three ovarian dermoid cysts, two localized in the same ovary. In a retrospective study, Hao et al. [14]. revealed that patients with mature teratoma experienced three bilateral recurrences within 10 years. Wang et al. [21,22]. analyzed the gene profile of teratomas and indicated that cystic teratomas originate from different stages of oocyte or primary oocyte before germinal vesicle stage failure of meiosis I in female gametogenesis. This provides evidence of metachronous development of ovarian mature teratoma, highlighting the importance of accurate preoperative examination and the necessity of regular postoperative follow-up.

The literature reports that the recurrence rate of benign ovarian tumors ranges from 2.6% to 12.1% [1,5,6,16,23]. Kiely et al. [7]. summarized in a systematic review that the recurrence rate of benign ovarian tumors increases with longer postoperative follow-up periods. Taskinen et al. [10]. followed up with 22 patients who underwent oophorectomy for mature teratomas for up to 25.5 years and found that 5 patients developed metachronous ovarian tumors, indicating a recurrence rate as high as 23%. Only a small number of studies report the specific recurrence time of benign tumors. In a multicenter retrospective study of 426 patients, 75% of those suspected of recurrence based on imaging developed recurrent lesions at 30.2 months [16]. In another retrospective study of 66 patients, 85.7% (6/7) of recurrent cases were detected one year after surgery [5]. Braungart et al. [6] revealed that 77.8% (7/9) of recurrent cases developed recurrent lesions within 2 years in their study involving 177 mature teratomas. In this study, 84.6% (11/13) of recurrent cases occurred within 3 years after surgery. Based on the above information, it can be concluded that the recurrence of benign ovarian tumors mainly occurs in the first 3 years after surgery. Therefore, the authors believe that a minimum follow-up period of 3 years is necessary.

In this study, non-neoplastic ovarian cysts were detected in 30 patients during follow-up, which led some parents to increase the frequency of outpatient visits and ultrasound examinations. Some authors oppose routine surveillance for asymptomatic patients on the grounds that the discovery of asymptomatic lesions leads to additional imaging or bloodwork for tumor markers, as well as unnecessary reoperation [11]. In response to this, the authors believe that adopting strict surgical indications can reduce or avoid unnecessary reoperation. For asymptomatic simple or hemorrhagic cysts with a maximum diameter less than 8 cm, conservative management is typically recommended, unless there is continuous enlargement or the development of symptoms that necessitate surgery [24, 25, 26].

## Conclusions

Ovary-sparing surgery for benign ovarian tumors has a favorable prognosis and a high rate of ovarian preservation. The authors believe that regular follow-up after surgery for benign ovarian tumors is necessary. Annual imaging follow-up for at least 3 years postoperative can detect most recurrence cases.

## Authors’ contributions

Design of the work; the acquisition, analysis and interpretation of data; draft manuscript:XiaoLi Chen.

Critically revise the manuscript and analysis of data: DuoTe Cai, Yi Chen, WeiWei Chen.

Critically revise manuscript: XiaoLi Chen, YueBin Zhang, QingJiang Chen, ZhiGang Gao.

## Conflicts of interest

The authors declare no conflicts of interest.
